# ﻿*Dendrocorticiopsisorientalis* gen. et sp. nov. of the Punctulariaceae (Corticiales, Basidiomycota) revealed by molecular data

**DOI:** 10.3897/mycokeys.90.84562

**Published:** 2022-05-31

**Authors:** Chia-Ling Wei, Che-Chih Chen, Shuang-Hui He, Sheng-Hua Wu

**Affiliations:** 1 Department of Biology, National Museum of Natural Science, Taichung 40453, Taiwan Department of Biology, National Museum of Natural Science Taichung Taiwan; 2 Department of Plant Pathology, National Chung Hsing University, Taichung 40227, Taiwan National Chung Hsing University Taichung Taiwan; 3 Biodiversity Research Center, Academia Sinica, Taipei 11529, Taiwan Academia Sinica Taipei Taiwan; 4 School of Ecology and Nature Conservation, Beijing Forestry University, Beijing 100083, China Beijing Forestry University Beijing China

**Keywords:** Corticioid fungi, East Asia, phylogeny, taxonomy, wood-decaying fungi

## Abstract

*Dendrocorticiopsisorientalis* is presented in this study as a new genus and new species based on morphological and phylogenetic evidence. This new taxon is characterized by resupinate, smooth and membranaceous basidiomata, monomitic hyphal system with clamps, colorless dendrohyphidia, variable presence of cystidia, and ellipsoid to ovoid basidiospores measuring 5–7 × 3.2–5.2 μm. The phylogenetic analyses based on the ITS1-5.8S-ITS2 (ITS) + nuclear 28S rDNA (28S) dataset of Corticiales indicated that the new taxon is nested in Punctulariaceae, separated from other genera with strong support values. Descriptions, specimen photo, and illustrations of the new taxon are provided in this study. A morphological comparison of the four genera of Punctulariaceae is given.

## ﻿Introduction

Corticiales K.H. Larss. is a small order of corticioid fungi with four families: Corticiaceae Herter, Dendrominiaceae Ghobad-Nejhad, Punctulariaceae Donk, and Vuilleminiaceae Maire ex Lotsy. The members of the order show a variety of nutritional ecologies, including lignicolous saprobes, foliicolous species, plant pathogens, and lichenicolous species (Ghobad-Nejhad et al. 2010, 2021). Species of Punctulariaceae are mainly saprobic on angiosperm trees, causing white rot. Morphologically, they are characterized by having effused to effused-reflexed basidiomata, smooth to tuberculate hymenial surface, a monomitic hyphal system with clamped generative hyphae, mostly absence of cystidia, sparsely to regularly branched dendrohyphidia, and ellipsoid to subglobose basidiospores which are negative in Melzer’s reagent and acyanophilous in cotton blue. When Donk (1964) established this family, he adopted Talbot’s suggestion (Talbot 1958) and designated *Punctularia* Pat. as the type genus. Ghobad-Nejhad et al. (2010) were the first to use a phylogenetic approach to analyze Punctulariaceae, and they recognized three genera, viz., *Dendrocorticium* M.J. Larsen & Gilb., *Punctularia*, and *Punctulariopsis* Ghobad-Nejhad. This arrangement was generally accepted by mycologists (Hibbett et al. 2014; He et al. 2019; Wijayawardene et al. 2020).

Most of the previous studies of Punctulariaceae focused on European species (Bernicchia and Gorjón 2010; Gorjón and Bernicchia 2017), although species from other continents received attention as well (Ghobad-Nejhad et al. 2010; Baltazar et al. 2013; Ariyawansa et al. 2015). However, the study of this family in Asia is insufficient and needs an update (Petch 1916; Cooke 1956; Guan et al. 2021). During surveys of corticioid fungi in East Asian regions, we found an unknown species morphologically similar to *Dendrocorticium* spp. Phylogenetic analyses were conducted by using ITS+28S sequences to evaluate the generic placement of the target taxon, and the results indicated that it represents a new genus and a new species of the Punctulariaceae.

## ﻿Materials and methods

### ﻿Morphological studies

Descriptions and illustrations are based on dried specimens deposited at the herbaria of the National Museum of Natural Science (**TNM**) and Beijing Forestry University (**BJFC**). Specimens were sliced into thin sections under stereo microscope (Nikon SMZ645) and mounted in 5% KOH with 1% phloxine in preparation for observations and measurements. Melzer’s reagent (IKI) and cotton blue were applied to detect amyloidity or dextrinoidity, and cyanophily, respectively. Microscopic studies were carried out under 1,000× magnification using an optical microscope (Olympus BX43). For presenting the range of basidiospore dimensions, 5% values of minimum and maximum are given in parentheses.

### ﻿DNA extraction and sequencing

DNA was extracted from dried specimens using the Plant Genomic DNA Extraction Miniprep System (Viogene Biotek corporation, New Taipei City, Taiwan), following the manufacturer’s protocol. ITS1-5.8S-ITS2 and partial 28S regions were amplified with the primer pairs ITS1/ITS4 (White et al. 1990) and LR0R/LR5 (Vilgalys and Hester 1990). The PCR protocols for ITS and 28S followed Chen et al. (2020). PCR products were purified and sequenced by MB Mission Biotech company (Taipei City, Taiwan). New sequences were assembled and adjusted using BioEdit v7.2.5 (Hall 1999) and subsequently submitted to GenBank (Table 1).

**Table 1.** Information of species and strains used in phylogenetic analyses, including their localities, voucher numbers, and GenBank accession numbers (ITS and 28S). Newly generated sequences are shown in bold. Voucher number of holotypes are marked with an asterisk (*).

Species	Locality	Voucher no.	GenBank accession no.	
			ITS	28S
*Australovuilleminiacoccinea* Ghobad-Nejhad & Hallenb.	New Zealand	PDD:94158*	HM046875	HM046930
*Basidiodeserticahydei*	Oman	DST2020a_SQUCC15289*	MW077150	MW077159
*Corticiumroseum*	China	Ghobad-Nejhad 2428	MW805872	MW805836
*C.thailandicum*	Thailand	Ghobad-Nejhad 3012	MW805868	MW805831
*Cytidiasalicina* (Fr.) Burt	Finland	Haikonen 24631	GU590881	HM046921
*Dendrocorticiopsisorientalis* Sheng H. Wu, C.L. Wei & S.H. He	Taiwan	WEI 20-166*	**MW580922**	**MW580924**
*D.orientalis*	Taiwan	WEI 20-173	**MW580925**	**MW580927**
*D.orientalis*	Taiwan	BCRC 36235	EU232219	EU232303
*D.orientalis*	China	He 4195	**MW580926**	**MW580921**
*Dendrocorticiumpolygonioides* (P. Karst.) M.J. Larsen & Gilb.	France	CBS 106.56	MH857525	MH869062
*D.roseocarneum* (Schwein.) M.J. Larsen & Gilb.	South Korea	KUC20121109-32	KJ668559	KJ668413
*Dendrominiadryina* (Pers.) Ghobad-Nejhad & Duhem	France	Duhem 5283	JX892936	JX892937
*D.ericae* (Duhem) Ghobad-Nejhad & Duhem	France	Duhem 4840*	JX892938	JX892939
*Disporotrichumdimorphosporum*	USA	CBS 433.85	MH861895	MH873584
*D.dimorphosporum*	Netherlands	CBS 419.70*	MH859776	MH871538
*Erythriciumhypnophilum*	France	MG169	MW805858	MW805823
*E.laetum*	—	Kotiranta 21287	GU590875	GU590878
*Gloeophyllumabietinum* (Bull.) P. Karst.	Switzerland	H 22988	JX524619	KC782733
*L.fuciformis*	Netherlands	CBS 182.49	MH856485	MH868023
*L.roseipellis*	—	CBS 299.82	EU622846	EU622844
‘*Lawreymycespalicei*’	—	Palice 4369*	AY542865	AY542865
‘*Lawreymycespalicei*’	—	Palice 2509	AY542864	AY542864
*Marchandiomycesaurantioroseus* (P. Karst.) Ghobad-Nejhad	Sweden	Hallenberg 8186	KP864659	HM046929
*M.corallinus*	—	JL128-98	AY583327	AY583331
*Mycobernardiaincrustans*	France	Duhem 3613	MW805860	MW805825
*M.incrustans*	Canada	CBS172.36	MH855759	MH867272
*Punctulariaatropurpurascens* (Berk. & Broome) Petch	Taiwan	WEI 17-662	**MW570883**	**MW570888**
*P.bambusicola* C.L. Zhao	China	CLZhao 9098*	MW559983	MW559985
*P.strigosozonata* (Schwein.) P.H.B. Talbot	—	HHB-11897-sp	DQ398958	AF518642
*Punctulariopsisefibulata* (M.J. Larsen & Nakasone) Ghobad-Nejhad	USA	Burdsall 8824*	KR494276	KR494277
*P.obducens* (Hjortstam & Ryvarden) Ghobad-Nejhad	Ethiopia	Ryvarden 28131	HM046918	HM046933
*P.subglobispora* (Hallenb. & Hjortstam) Ghobad-Nejhad	Argentina	Hallenberg 12761*	HM046917	HM046932
*Veluticepsabietina* (Pers.) Hjortstam & Tellería	Sweden	KHL 12474	EU118619	EU118619
*Vuilleminiacomedens* (Nees) Maire	—	T-583	DQ398959	AF518666
*V.coryli* Boidin, Lanq. & Gilles	Turkmenistan	Parmasto 54999	JN387996	JN388005
*V.cystidiata* Parmasto	South Korea	KUC20131022-26	KJ668433	KJ668285
*V.erastii* Ghobad-Nejhad	Canada	DAOM 199025*	JN387998	JN388007
*V.macrospora* (Bres.) Hjortstam	France	Duhem 4860	JX892940	JX892941
*V.megalospora* Bres.	Italy	Ryvarden 43185	HM046887	HM046926
*V.nilsii* Ghobad-Nejhad & Duhem	France	Duhem 4847*	JX892947	JX892948
*V.pseudocystidiata* Boidin, Lanq. & Gilles	France	Boidin 14838*	HM046888	HM046928
*Waiteacircinata*	USA	CBS472.82	MH861518	MH873265
*W.guianensis*	French Guiana	GUY13-110	MW449090	MW449101

### ﻿Phylogenetic analyses

The selection of species and samples for the ITS+28S dataset was inspired by Ghobad-Nejhad and Duhem (2014) and Guan et al. (2021). The dataset contained 43 samples from 37 species, including 35 ingroup species from 17 genera of the four families in Corticiales and 2 outgroup species from Gloeophyllales [*Gloeophyllumabietinum* (Bull.) P. Karst. and *Veluticepsabietina* (Pers.) Hjortstam & Tellería, Table 1]. Sequences were aligned in MAFFT v.7 (Katoh and Standley 2013). Partitioned phylogenetic analyses were carried out for the ITS+28S dataset based on maximum likelihood (ML) and Bayesian inference (BI) methods, using MrBayes v. 3.2.6. (Ronquist et al. 2012) and RaxML Black Box (Stamatakis 2014) at the CIPRES Science Gateway (http://www.phylo.org/). For the BI analysis, jModeltest 2.1.10 (Darriba et al. 2012) was first executed to estimate the best-fit substitution model based on Akaike Information Criterion (AIC). The GTR+G+I was used as the substitution model for the ITS1, ITS2 and 28S regions, while K80 was used for 5.8S region. The parameter settings for ML and BI analyses followed Wu et al. (2018). Only the phylogram inferred from the ML analysis is shown since the BI and ML analyses produced similar topologies. The statistical support values are presented above the branches of the ML tree when bootstrap values (BS) ≥ 70 and BI posterior probability (PP) ≥ 0.9. The complete phylogenetic trees and alignment were submitted to TreeBASE (submission number 29602; www.treebase.org).

## ﻿Results

### ﻿Phylogenetic inference

**Figure 1.**
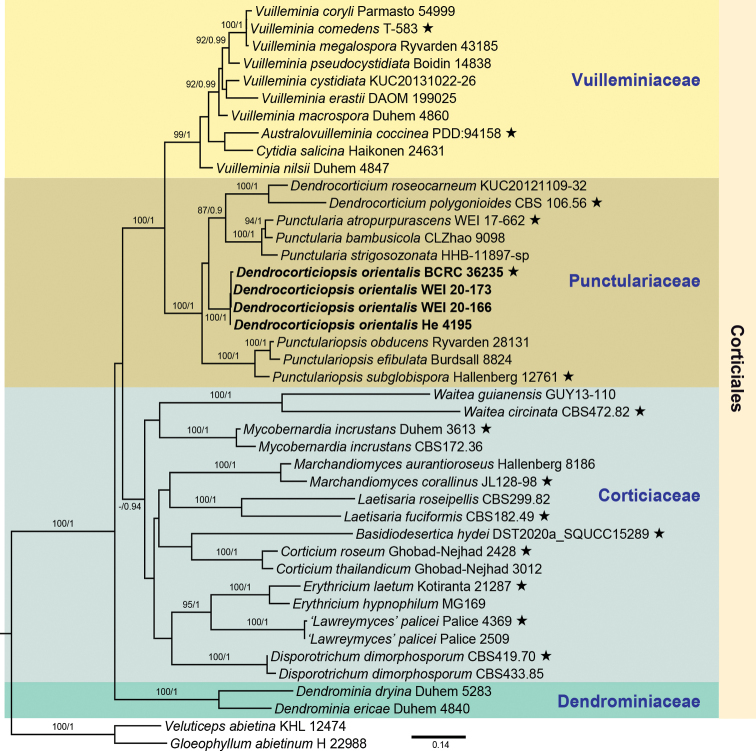
The phylogram of Corticiales inferred from ML analysis using the combined ITS+28S dataset shows the position of *Dendrocorticiopsisorientalis* (shown in bold) in Punctulariaceae. Numbers above branches indicate statistical support of BS ≥ 70% and PP ≥ 0.9. Black stars (é) indicate strains of generic species.

The final alignment of 43 sequences contained 1,647 sites (including gaps) of which 724 sites were from the ITS region and 923 sites from the 28S gene. Totally, 565 (34%) sites were parsimony informative. The ML tree (Fig. 1) shows the four highly supported families also recovered in previous studies (Ghobad-Nejhad and Duhem 2014; Ariyawansa et al. 2015; Ghobad-Nejhad et al. 2021; Guan et al. 2021). The four samples of the new species *Dendrocorticiopsisorientalis* formed a monophyletic group in Punctulariaceae with strong support values (BS = 100%; PP = 1), well separated from the other genera, viz., *Dendrocorticium*, *Punctularia*, and *Punctulariopsis* (Fig. 1). Therefore, *Dendrocorticiopsis* is treated as the fourth genus of Punctulariaceae.

### ﻿Taxonomy

#### ﻿ Dendrocorticiopsis

Taxon classificationFungiCorticialesPunctulariaceae

Sheng H. Wu, C.L. Wei & S.H. He gen. nov.

2379585E-A4DD-530F-A547-2CA1F22AE429

MycoBank: MB838902

##### Diagnosis.

*Dendrocorticiopsis* differs from other genera by having strictly resupinate basidiomata, ivory hymenphore, a compact texture, a monomitic hyphal system, nodose-septate hyphae, encrusted cystidia, dendrohyphidia and ellipsoid to ovoid basidiospores.

##### Description.

Basidiomata resupinate, effused, adnate, membranaceous. Hymenial surface brownish ivory, grayish ivory to lilac ivory, smooth. Hyphal system monomitic; generative hyphae nodose-septate, colorless, slightly thick- to thick-walled. Subiculum uniform, with compact texture, usually with crystal masses; hyphae fairly horizontal. Hymenial layer thickening, with compact texture, usually with oily materials, hyphae more or less vertical. Dendrohyphidia numerous, thick-walled toward base, colorless. Cystidia clavate, apically with resinous materials. Basidia clavate to subclavate, 4-sterigmata, thick-walled toward base. Basidiospores ellipsoid to ovoid, sometimes broadly ellipsoid, smooth, thin-walled or occasionally slightly thick-walled, negative in Melzer’s reagent, acyanophilous.

##### Type species.

*Dendrocorticiopsisorientalis*.

##### Etymology.

*Dendrocorticiopsis* refers to the morphological resemblance to *Dendrocorticium*.

#### ﻿ Dendrocorticiopsis orientalis

Taxon classificationFungiCorticialesPunctulariaceae

Sheng H. Wu, C.L. Wei & S.H. He sp. nov.

82EEBE91-A113-5B1F-9DA2-2FAFF8228249

MycoBank: MB838903

Figs 2 , 3

##### Diagnosis.

The noteworthy features of *Dendrocorticiopsisorientalis* are: (1) subiculum composed of a basal layer, with compact texture; (2) oily materials usually present in hymenial layer; (3) cystidia with resinous materials at apices; (4) shortly clavate to subclavate basidia; (5) ellipsoid to ovoid basidiospores measuring 5–7 × 3.2–5.2 μm.

**Figure 2.**
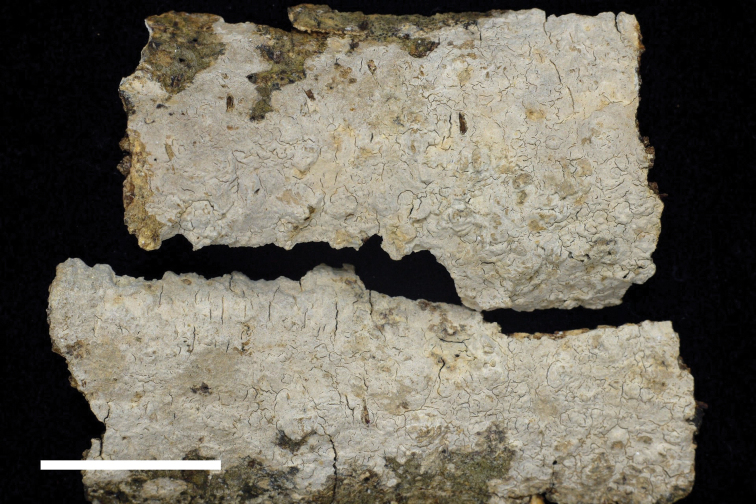
Basidiomata of *Dendrocorticiopsisorientalis* (holotype, WEI 20-166). Scale bar: 1 cm.

##### Typification.

Taiwan, Taichung City, Heping District, near trailhead of Mt. Tangmadan Trail, 24°09'53.0"N, 120°57'26.4"E, 670 m asl., on dead angiosperm trunk, 20 Aug 2020, leg. C.L. Wei, WEI 20-166 (holotype, TNM F34448). GenBank: ITS = MW580922; 28S = MW580924.

**Figure 3.**
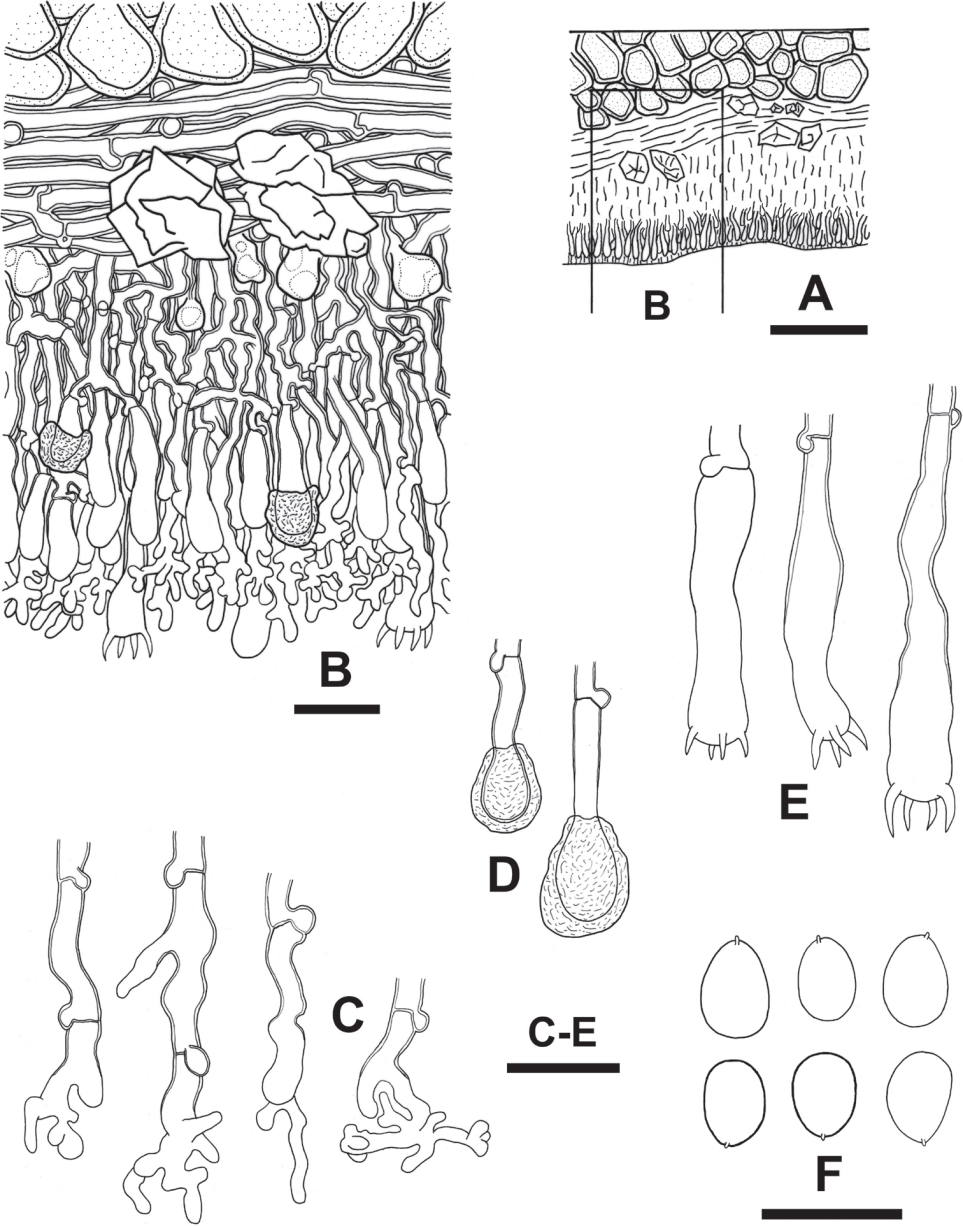
Micromorphological features of *Dendrocorticiopsisorientalis* (holotype, WEI 20-166) **A** profile of basidioma section **B** basidioma section **C** dendrohyphidia **D** cystidia **E** basidia **F** basidiospores. Scale bars: 50 μm (**A**); 10 μm (**B–F**).

##### Etymology.

The epithet refers to the Eastern world, where the specimens were collected.

##### Description.

Basidiomata annual, resupinate, effused, adnate, membranaceous, 50–100 μm thick in section. Hymenial surface brownish ivory, grayish ivory to lilac ivory, smooth, finely cracked; margin concolourous, slightly pruinose, rather determinate. Hyphal system monomitic; generative hyphae nodose-septate. Subiculum fairly uniform, composed of a basal layer, with fairly compact texture, usually with crystal masses; up to 30 μm thick, sometimes indistinct; hyphae mainly horizontal, colorless, fairly straight, 3–4 μm diam, with walls slightly thickened up to 1 μm. Hymenial layer thickening, with more or less compact texture, usually with oily materials, 50–70 μm thick; hyphae more or less vertical, colorless, 2–4 μm diam, with walls slightly thickened up to 1 μm. Dendrohyphidia numerous, 12–28 × 2–3 μm, thick-walled toward base, with walls up to 1 μm thick, colorless. Cystidia clavate, apically with resinous materials, gradually dissolving in KOH, 10–20 × 3.5–5.5 μm, slightly thick-walled, or thickening toward base, with walls up to 1 μm thick. Basidia clavate to subclavate, usually broadened at basal or middle parts, 18–35 × 5–7 μm, 4-sterigmata, thickening toward base, with walls up to 1 μm thick. Basidiospores ellipsoid to ovoid, or broadly ellipsoid, smooth, colorless, with homogenous contents, thin-walled or occasionally slightly thick-walled, negative in Melzer’s reagent, acyanophilous, mostly 5–7 × 3.2–5.2 μm. (5.5)6–7(7.5) × 4.2–5.2(5.5) μm, L = 6.50±0.42 μm, W = 4.66±0.32 μm, Q = 1.40 (n = 30) (holotype, WEI 20-166). (5.7)6.2–7(7.5) × (4.2)4.5–5(5.2) μm, L = 6.61±0.43 μm, W = 4.77±0.25 μm, Q = 1.39 (n = 30) (WEI 20-173). (4.2)5–6.8(7) × (3)3.2–5(5.2) μm, L = 5.8 μm, W = 4.2 μm, Q = 1.38 (He 4195).

##### Habitat.

On dead angiosperm wood (e.g., *Acacia* and *Castanopsis*), occurring in August.

##### Distribution.

In subtropical regions, known from China: Jiangxi and Taiwan.

##### Additional specimens examined (paratypes).

China, Jiangxi Province, Yichun City, Yifeng County, Guanshan National Nature Reserve, 500 m asl., on dead *Castanopsis* wood, 9 Aug 2016, leg. S.H. He, He 4195 (BJFC 023637). Taiwan, Taichung City, Heping District, near trailhead of Mt. Tangmadan Trail, 24°09'53.0"N, 120°57'26.4"E, 670 m asl., on dead angiosperm trunk, 20 Aug 2020 leg. C.L. Wei, WEI 20-173 (TNMF0034449).

##### Notes.

Both of the ITS and 28S sequences BLAST results showed that *Dendrocorticiopsisorientalis* is close to the strain BCRC 36235 that is annotated as *Ganodermaapplanatum* (Pers.) Pat. in GenBank. According to personal communication with Bioresource Collection and Research Center (BCRC, Taiwan), the strain BCRC 36235 was indeed isolated from a *Ganoderma* specimen collected by Dr. Jin-Torng Peng in Nantou, Central Taiwan, on wood of *Acaciaconfusa* Merr. However, as suggested by Suldbold (2017), the ITS (EU232219) and 28S (EU232303) sequences of the strain BCRC 36235 are not true *G.applanatum*, and we supposed that the strain could be contaminated by *D.orientalis*, which is known to grow on *Acacia*. The specimen He 4195 collected on *Castanopsis* (Fagaceae) from Jiangxi Province has slightly smaller basidiospores (L = 5.8 μm, W = 4.2 μm) than the holotype.

## ﻿Discussion

A comparison of morphological characteristics for distinguishing the four genera in Punctulariaceae is provided in Table 2. *Dendrocorticiopsis* is morphologically similar to *Dendrocorticium*, however, the latter has longer and narrowly clavate to tubular basidia usually longer than 45 μm, whereas *Dendrocorticiopsis* has clavate to subclavate basidia shorter than 35 μm. *Punctularia* differs from *Dendrocorticiopsis* by having resupinate or effused-reflexed basidiomata with a tuberculate hymenophore, colored dendrohyphidia, and through its lack of cystidia, while *Punctulariopsis* can be distinguished from *Dendrocorticiopsis* by possessing longer basidia and basidiospores, and mostly lacking cystidia.

**Table 2.** Morphological characteristics used for distinguishing the four genera in Punctulariaceae.

	*Dendrocorticiopsis*	*Dendrocorticium*	*Punctularia*	*Punctulariopsis*
**basidiomata**	resupinate	resupinate or effused-reflexed	resupinate or effused-reflexed	resupinate
**hymenial surface**	smooth	smooth	tuberculate	smooth
**dendrohyphidia**	colourless	mostly colourless (yellowish in *D.roseolum*); some species with encrustations	yellowish to brown or pink to rose	colourless
**cystidia**	clavate, apically with resinous materials	mostly absent (*D.roseolum* with halocystidia; *D.piceinum* with leptocystidia)	absent	mostly absent (*P.obducens* with leptocystidia)
**basidia**	clavate to subclavate; < 35 μm long	narrowly clavate to tubular; mostly > 45 μm long	narrowly clavate to tubular; 35–45 μm long	narrowly clavate to tubular; > 45 μm long
**basidiospores**	ellipsoid to ovoid; < 10 μm long	broadly ellipsoid to subglobose; usually < 10 μm long	ellipsoid; < 10 μm long	broadly ellipsoid to subglobose; > 10 μm long
**distributions**	subtropical regions	temperate or tropical regions	tropical to subtropical regions	tropical to subtropical regions

*Dendrocorticiumviolaceum* H.S. Jacks. ex M.J. Larsen & Gilb. and *D.polygonioides* (P. Karst.) M.J. Larsen & Gilb. have similar-sized basidiospores to *Dendrocorticiopsisorientalis* [4–6.5 × 3–5 μm in *D.violaceum*, 6–9 × 4–6 μm in *D.polygonioides* (Larsen and Gilbertson 1977)]. However, *D.violaceum* is distributed in Canada, has a reflexed basidiomata margin (closely adnate in *Dendrocorticiopsisorientalis*), and grows mainly on deciduous wood. *Dendrocorticiumpolygonioides* is mainly distributed in Europe and has a whitish to violaceous surface, large basidia (50–60 × 5–7 μm), and usually encrusted dendrohyphidia (Larsen and Gilbertson 1977).

## Supplementary Material

XML Treatment for Dendrocorticiopsis

XML Treatment for Dendrocorticiopsis orientalis

## Supplementary materials

Supplementary material 1 Alignments to TreeBase Data type Alignments (fas. file) Explanation note We have uploaded the alignments to TreeBase and here is the link and the file. http://purl.org/phylo/treebase/phylows/study/TB2:S29602?x-access-code=cdd27042a420e43e26dd8e62ea382799&format=html. https://binary.pensoft.net/file/690446This dataset is made available under the Open Database License (http://opendatacommons.org/licenses/odbl/1.0/). The Open Database License (ODbL) is a license agreement intended to allow users to freely share, modify, and use this Dataset while maintaining this same freedom for others, provided that the original source and author(s) are credited.Chia-Ling Wei, Che-Chih Chen, Shuang-Hui He, Sheng-Hua Wu
